# Tobacco Users

**Published:** 1882-06

**Authors:** 


### TOBACCO-USERS.

It has become very common to invest chewing-tobacco and snuff in lead-foil. Herr Hockel examined some snuff from a quantity, part of which had been used by a patient who was laboring under a severe attack of lead poisoning, and found that it contained two and a half per cent of metallic lead. The tobacco near the corners of the package, being more perfectly inclosed by the foil, contained the most lead, which is decomposed by dampness and remains in the tobacco or snuff in the form of carbonate of lead, which is the white lead paint of commerce, which inflicts such horrible sufferings on many of those whose business compels them to work in it. The slaves of the disgusting "weed" would do well to make a note of this, and either abandon the inexcusable filthiness or avoid using any that is enveloped with lead-foil.

### THE LONGEST LIVERS

Are they who dwell in Palaces and Poor Houses. As contradictory, as this appears, it is not the less true. The reason of it is, in the fact, that, knowing they are provided for, the mind is at rest, and is wholly disencumbered of that eating anxiety, that care for to-morrow, which press so heavily upon the mass of mankind. The very rich and the very poor are not the healthiest; on the contrary, they are seldom entirely well; this indisposition takes away what little appetite a loafing life allows them, hence, for a short time, they eat almost nothing: this gives the stomach time to recuperate, while nature works off the surplusage, and by this double operation, they are made as well as ever in a few days. Hence, the best "Life Insurance" is to secure for yourself, at the earliest possible day, *a moderate, uniform, and* CERTAIN *income.*

